# Epigenetic Mechanisms in DNA Double Strand Break Repair: A Clinical Review

**DOI:** 10.3389/fmolb.2021.685440

**Published:** 2021-07-07

**Authors:** Alejandra Fernandez, Connor O’Leary, Kenneth J O’Byrne, Joshua Burgess, Derek J Richard, Amila Suraweera

**Affiliations:** ^1^ Centre for Genomics and Personalised Health, School of Biomedical Sciences and Translational Research Institute, Queensland University of Technology (QUT), Brisbane, QLD, Australia; ^2^ Princess Alexandra Hospital, Woolloongabba, QLD, Australia

**Keywords:** DNA double strand breaks, DNA repair, epigenetic mechanisms, histone deacetylase inhibitors, DNA methyltransferase inhibitors

## Abstract

Upon the induction of DNA damage, the chromatin structure unwinds to allow access to enzymes to catalyse the repair. The regulation of the winding and unwinding of chromatin occurs via epigenetic modifications, which can alter gene expression without changing the DNA sequence. Epigenetic mechanisms such as histone acetylation and DNA methylation are known to be reversible and have been indicated to play different roles in the repair of DNA. More importantly, the inhibition of such mechanisms has been reported to play a role in the repair of double strand breaks, the most detrimental type of DNA damage. This occurs by manipulating the chromatin structure and the expression of essential proteins that are critical for homologous recombination and non-homologous end joining repair pathways. Inhibitors of histone deacetylases and DNA methyltransferases have demonstrated efficacy in the clinic and represent a promising approach for cancer therapy. The aims of this review are to summarise the role of histone deacetylase and DNA methyltransferase inhibitors involved in DNA double strand break repair and explore their current and future independent use in combination with other DNA repair inhibitors or pre-existing therapies in the clinic.

## Introduction

According to the World Health Organization (WHO), approximately 9.6 million people were estimated to die from cancer in 2018. The WHO defines cancer as an event involving abnormal cell growth that can occur in any part of the body and later invade adjoining sections or spread to other organs (World Health Organization, 2019). Cancer develops at a molecular level and requires specific management strategies to achieve efficient treatment (Hanahan and Weinberg, 2000). This genomic disease often results as a consequence of normal cellular processes. For example, events such as DNA double strand breaks (DSBs), which have been classified as the most detrimental damage to DNA, usually occur in the chromosome due to environmental exposure to irradiation, ultraviolet light, or other chemical agents. These adverse genomic breakages can lead to imbalanced expression of proteins that are crucial for genomic stability (e.g., BRCA1/2, TP53, RAD51C). However, DSBs can be repaired by either one of the two conserved DSB repair pathways; homologous recombination (HR) and non-homologous end joining (NHEJ) (Mavaddat et al., 2013; Brown et al., 2017).

Changes in an organism can also be caused by modifications of gene expression, rather than alterations in the genetic code itself, a phenomenon defined as epigenetics. The epigenome comprises chemical compounds that are usually inherited, but that can also be altered by environmental influences such as diet and pollutants. These epigenetic modifications are described as a chemical layer on top of the DNA, which influences the way cells read genes. For instance, epigenetic modifications play a significant role in regulating several cellular processes involved in DNA damage/repair and thus, influence transcription, DNA damage response signaling and genomic stability, which are all hallmarks of cancer.

With the purpose of understanding the many genetic abnormalities that comprise cancer as a disease, epigenetics has been shown to be involved in altered gene function and malignant cellular transformation in the development of both solid tumors and hematological malignancies (Brower, 2011; Maeda et al., 2018; Rosenquist et al., 2018). The major epigenetic modifications involved in gene regulation are histone tail modifications, DNA methylation, chromatin remodeling and post translational ATP-dependent modifications, such as small non-coding RNA expression and gene imprinting (Wilson et al., 2010; Chervona and Costa, 2012; Werner et al., 2017; Zhou et al., 2020; Alexandrova et al., 2020; Zhou et al., 2018).

Histone tail modifications involving deacetylation and DNA methylation are the two epigenetic modifications most widely explored to date. Histone deacetylation is catalyzed by histone deacetylases (HDACs). On the other hand, DNA methylation is catalyzed by DNA methyltranferases (DNMTs) (Tu et al., 2020; Narayan et al., 2020; Ghasemi, 2020; Li et al., 2018; Mazzone et al., 2017; Werner et al., 2017; Villanueva et al., 2020). These discoveries have paved the way for targeted epigenetic therapy used in the clinic for the treatment of cancer. The inhibition of histone deacetylation and DNA methylation epigenetic mechanisms are a highly desirable target for novel drugs. The U.S Food and Drug Administration (FDA) has approved histone deacetylase inhibitors (HDACi) and DNA methyltransferase inhibitors (DNMTi) that are currently being used independently, or in combination with other cancer therapies (Narayan et al., 2020; FDA, 2020a; Mann et al., 2007). In this review, we investigate the mechanisms and effects of HDAC-HDACi and DNMT-DNMTi in DSB repair and their impact and/or potential as therapeutic agents.

## Cancer, DNA Damage and Epigenetic Changes

### DNA Damage as a Hallmark of Cancer

DNA damage has been defined as a hallmark of cancer (Hanahan and Weinberg, 2000; Andor et al., 2017). To remain guarded, the genome is reliant on stable DNA damage responses (DDR). Depending on the type of DNA damage, a signaling network is activated upon the detection of the DNA lesion to coordinate DNA repair, the cell cycle, senescence or apoptosis, in order to restore the genetic information (Falck et al., 2005). Hence, cancer cells can develop dysfunctional DNA repair mechanisms which further promote oncogenesis; however, this genomic instability can be exploited in cancer therapy (Jeggo and Löbrichf, 2015; Sokol et al., 2020; Caracciolo et al., 2021).

DNA damage may also lead to failures in cell cycle checkpoint activation, dysfunctional redox homeostasis and telomere attrition (Gad et al., 2014; Huber et al., 2014). Despite DNA being able to easily repair such lesions through DNA repair mechanisms, when these processes fail, mutations occur and this can predispose individuals to cancer (Hanahan and Weinberg, 2000; Andor et al., 2017). There are different types of DNA damage, including abasic sites (DNA base is missing), mismatches (replication errors), modified bases (changes to the bases), inter-strand crosslinks (covalent linkage between the two strands), single-strand breaks (a break in the sugar-phosphate backbone of one strand) or DSBs (both strand backbones are broken) (Ward, 1985; Vaz et al., 2017). A multiplicity of DNA repair systems has evolved in order to counteract these lesions. Some of these repair mechanisms involve base excision repair (BER), mismatch repair (MMR), post-replication repair and error-prone repair systems (Iyama and Wilson, 2013; Vaz et al., 2017; Rajapakse et al., 2019). BER features three steps: excision of the damaged base; use of the undamaged DNA strand as a template to fill in the gap via DNA polymerase; and DNA ligase to seal the process (Sancar, 1994). MMR, proofreads and corrects mismatched nucleotides (Kunkel and Erie, 2005). Post-replication repair involves modification of existing gaps in newly synthesized strands. The two most predominant post-replication repair systems are translation synthesis and template switching (Kaufmann, 1989). Lastly, homologous recombination (HR) and non-homologous recombination (NHEJ) pathways are involved in DSB repair, the most cytotoxic type of DNA backbone damage (Rodgers and Mcvey, 2016), which is discussed in more detail below.

### DNA Double Strand Breaks

**FIGURE 1:**
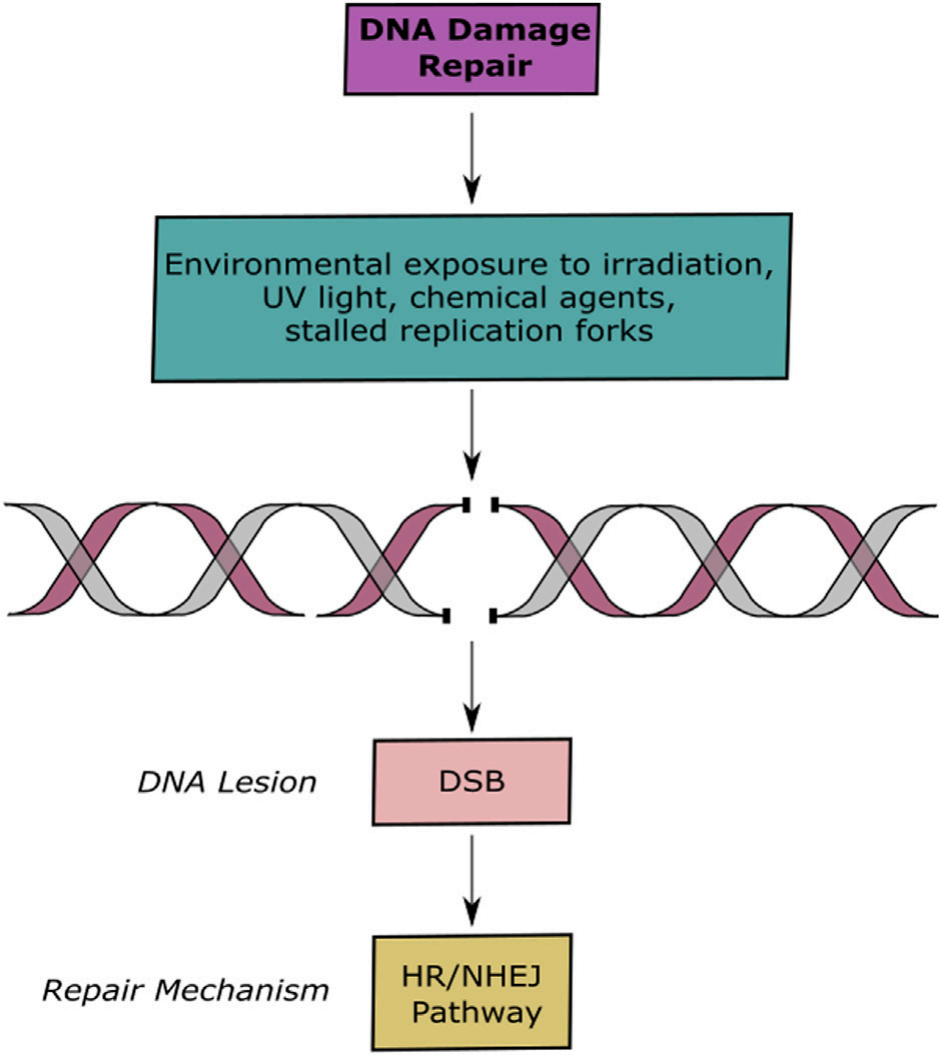
DNA Lesions and Repair Pathways. Schematic representation of DNA damage and repair. Exogenous and endogenous agents induce DSBs, which are repaired by the HR or NHEJ pathways.

In contrast to single strand breaks, DSBs involve the breakage of the two strains of the double helix, making it more difficult to repair. These lesions bring alongside severe mutagenic consequences that promote oncogenesis. DNA DSBs occur spontaneously or are caused by both exogenous and endogenous agents (Takata et al., 1998; Moroni et al., 2013; Moloney and Cotter, 2018; Schwartz et al., 2005) (Figure 1). In response to this genetic insult, cells have evolved to recognize the damage and signal for DNA DSB repair mechanisms. The proteins responsible of signaling these events are PIKKs (phosphatidykinositol 3-kinase-related kinases), DNA-PKcs (DNA-dependent serine/threonine protein kinase catalytic subunit), ATM (ataxia telangiectasia mutated), and ATR (ataxia telangiectasia and Rad3-related protein). Unrepaired or incorrect repaired DSBs often lead to the loss of genetic information, chromosomal aberrations, unregulated cell division or cell death proceeding with genomic instability, which is a hallmark of cancer (Johnston et al., 1961; Antonarakis et al., 2004; Jackson and Bartek 2009; Jekimovs et al., 2014).

### DNA Double Strand Break Repair Mechanisms

The two most conserved repair pathways are homologous recombination (HR) and non-homologous end joining (NHEJ) (Schwartz et al., 2005; Takata et al., 1998; Essers et al., 2000). These two pathways work collaboratively but can also compete with each other (Figure 1) (Decottignies, 2013). Cells undergo a regulated mechanism to choose between these two pathways, the progressive 5–3′ resection of DNA ends promotes HR dependent repair and blocks NHEJ (Escribano-Díaz et al., 2013). On the other hand, binding of the Ku70/Ku80 complex enables repair of the damage site via NHEJ by protecting DNA ends from exonucleases and by preventing HR pathway mechanisms. Additionally, it has been reported that RIF1 and 53BP1 play an important role in promoting NHEJ mechanisms, while, BRCA1 and RBBP8 promote HR mechanisms (Chapman et al., 2012; Escribano-Díaz et al., 2013).

#### Homologous Recombination

**FIGURE 2:**
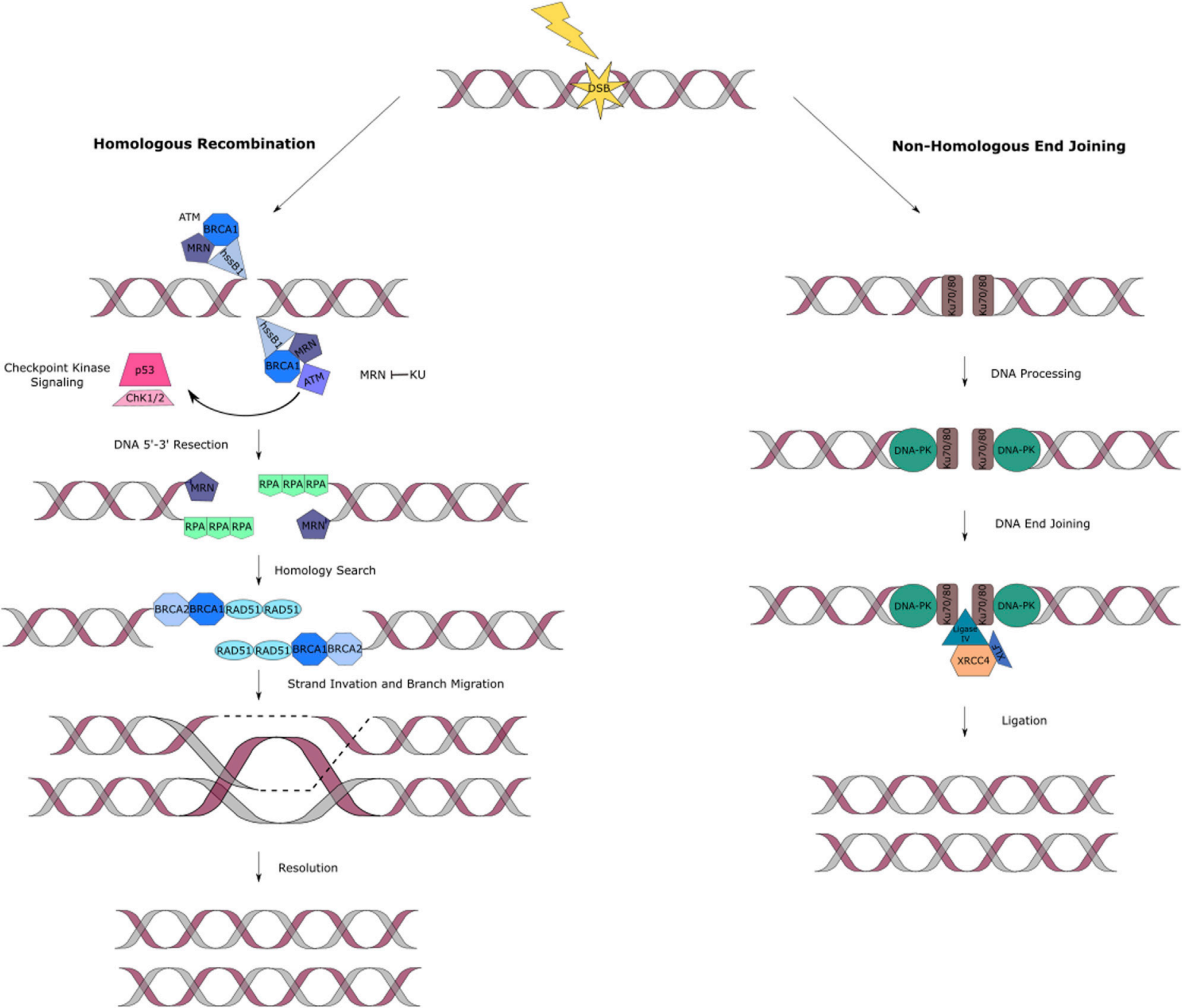
DNA double strand break repair pathways. **(A)** HR fixes two-ended DSBs by a resection process. A recombinase will then induce strand invasion. The single strand is then extended, using the complementary strand as template. Recapture of the second end occurs followed by ligation. The main proteins involved in this pathway are hSSB1, MRN complex, RPA, BRACA1/2 and Rad51. **(B)** NHEJ of DSBs in DNA is accomplished by a series of proteins that work together to carry out the synapsis, preparation, and ligation of the broken DNA ends. The main proteins involved in NHEJ eukaryotes are Ku and DNA-PK complexes, XLF and the XRCC4/DNA ligase IV complex.

HR is a strand invasion mechanism that occurs during the late S to G2 phase of the cell cycle and is known to be unerring as it uses the presence of a homologous chromosome or sister chromatid as a template for the repair (Figure 2A) (Essers et al., 2000). Human single stranded DNA binding protein 1 (hSSB1) has shown to be an essential protein to signal for DNA DSB repair through HR by recruiting the MRN (Mre11/Rad50/NBS1) complex to the lesion site (Lawson et al., 2020; El-Kamand et al., 2020; Ashton et al., 2017; Croft et al., 2017; Touma et al., 2016; Paquet et al., 2016; Richard et al., 2011a; Richard et al., 2011b; Richard et al. 2008). The MRN complex is responsible for activating the ATM kinase activity and binding the DNA ends at the break site (D’Amours and Jackson 2002). This complex also plays an important role in the DSB repair pathway selection. This occurs depending on the cell type, cell cycle stage and by competing with the binding of the Ku70/80 complex, which favors NHEJ, at the damage site (Lamarche et al., 2010). Once HR has been selected as the pathway to proceed with, for lesion repair, the ATM kinase initiates a cascade of events that signal for DSB resection to produce single-stranded DNA (ssDNA), that later acts as a substrate for recombinase Rad51 (Jazayeri et al., 2008). The process continues with resection of the DNA by exposing the ssDNA through the binding of replication protein A (RPA) (Garcia et al., 2011; Tomimatsu et al., 2012). RPA also aids in protecting DNA from inappropriate annealing that could alter the genome (Bolderson et al., 2010). BRCA1 ensures that RPA remains bounded to the lesion site (Chen et al., 2008). BRCA2 removes RPA exposing ssDNA and stimulating the activity of the Rad51. Rad51 creates a helical filament on ssDNA which hunts for nearby homologous double-stranded DNA facilitating strand invasion of the sister chromatid to finally repair the damage site. The final stage is resolution of the Holliday junction and ligation of the broken phosphate backbone (Figure 2A) (Yuan et al., 1999; Helleday et al., 2007; Jekimovs et al., 2014).

#### Non-homologous End Joining

The NHEJ (also known as classical non-homologous or C-NHEJ) pathway takes place during all cell cycle stages, where it repairs DSBs through direct ligation (Figure 2B). NHEJ is the only available pathway in the G0 to G1 phases of the cell cycle. In contrast to HR, it does not use a homologous sister chromatin to fix DSBs, making it a potentially error-prone mechanism (Shrivastav et al., 2008; Jekimovs et al., 2014).

NHEJ follows a system involving recognition of the damage at site, DNA processing and ligation. Ku (Ku70 and Ku80 heterodimers) and DNA-PK are the most relevant protein complexes involved in this pathway (Dobbs et al., 2010). Ku recognizes the DNA DSB and it is responsible for protecting the DSB ends from degradation and attack of exonucleases. Similarly, it is in charge of recruiting other DNA damage repair proteins (Takata et al., 1998). DNA-PKcs, is a holoenzyme which functions to link the DNA ends together and DNA-PK is auto-phosphorylated either before or after the processing stage (Boldogh et al., 2003). These ends are processed by enzymes like the MRN complex. The DNA damage repair is finalized by stimulating end-joining. This occurs by the interaction of XLF and the XRCC4/DNA ligase IV complex (Figure 2B) (Ahnesorg et al., 2006).

### Histone Acetylation and DNA Methylation as Epigenetic Regulator Mechanisms Involved in DNA Double Strand Break Repair

**FIGURE 3:**
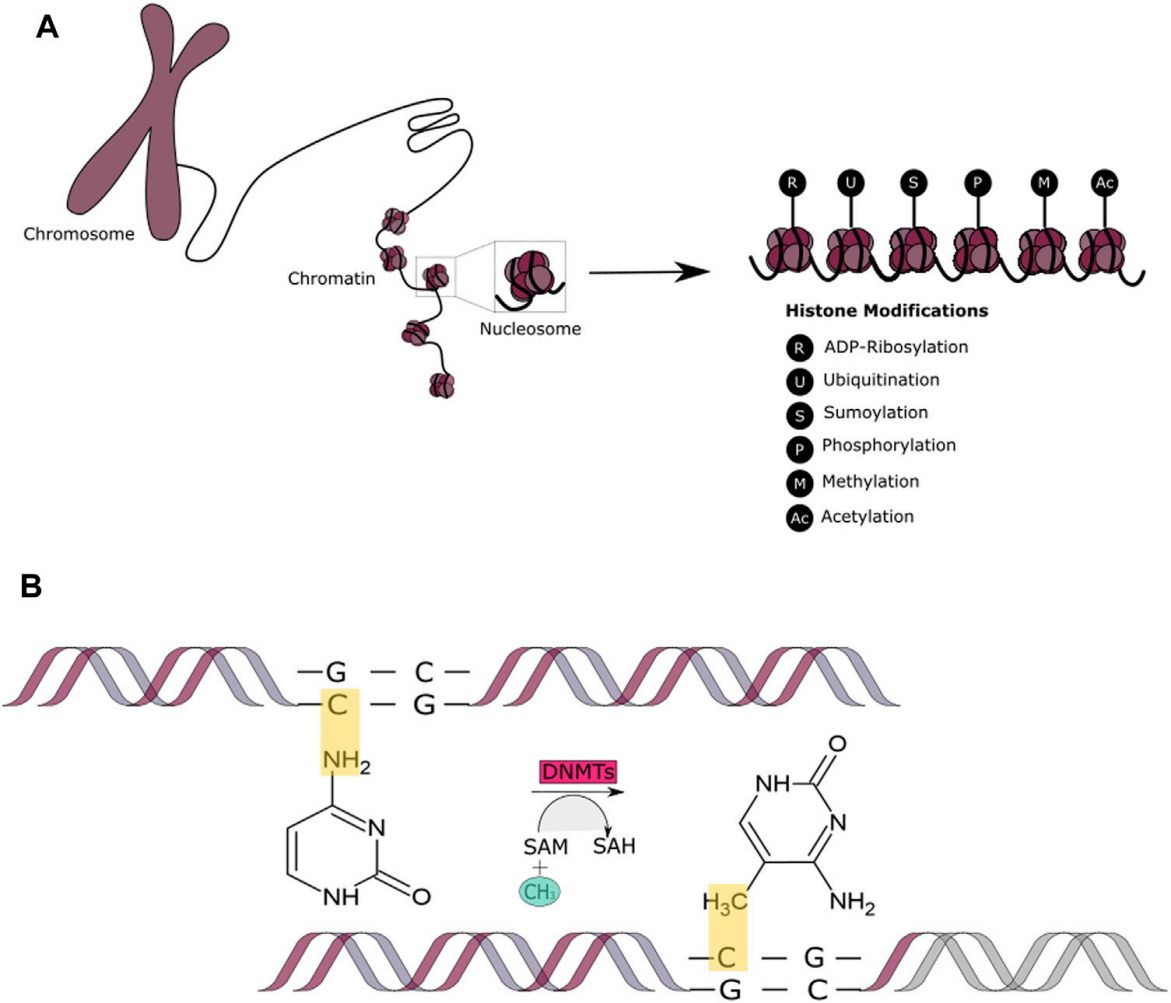
Epigenetic Mechanisms - Histone Modifications and DNA methylation. **(A)** A schematic representation of the covalent post-translational modifications to histone proteins. These include ADP-ribosylation, ubiquitination, sumoylation, methylation, acetylation, and phosphorylation. **(B)** A schematic representation of the DNA methylation process that occurs by addition of the methyl (CH_3_) group to the DNA, thereby often modifying the function of certain genes and affecting gene expression.

DNA is wrapped around histone proteins that are grouped into nucleosomes, which are coiled into a fiber that is later condensed into chromatin. When histones are modified, they affect gene expression regulation, protein activation and stability and can also enable or disable the access of transcription factors to the nucleotides (Mercurio et al., 2012) (Figure 3A). This can occur via epigenetic events known as histone modifications that are catalyzed through enzymatic activities that trigger reversible post-translational modifications such as: ADP-ribosylation (modification of histone ribosylation sites Aspartic/Glutamic acid) (Karch et al., 2017); ubiquitination (addition of a ubiquitin protein usually in histone H2A, lysine 119, and histone H2B, lysine 120) (Mercurio et al., 2012); sumoylation (addition of a small ubiquitin-related modifier SUMO, 11 kDa protein, at a lysine site) (Nathan et al., 2003); phosphorylation (mostly occurs in histone H2A(X), also known as γH2AX, at serine 139) (Jeggo and Löbrichf, 2015; Nair et al., 2017); methylation (a methyl group is added to a lysine or arginine residue in the histone tails) (Gupta et al., 2016); or acetylation.

However, in this review, we are mainly focusing on histone acetylation and DNA methylation as these have been the most widely studied epigenetic mechanisms due to their ability to modify chromatin and regulate transcriptional activity (Shinjo and Kondo 2015; Thakore et al., 2015; Podolsky et al., 2016). It has also been shown that histone modifications such as histone deacetylation and histone methylation can interact with DNA methylation to achieve long-term transcriptional repression (Freitag and Selker, 2005). It is important to mention that the deregulation of either of these epigenetic mechanisms during cancer initiation or progression can lead to resistance to therapy (Emran et al., 2019; Zhu et al., 2019).

Histone acetylation occurs through the addition of an acetyl group via acetyl-CoA to a lysine site at the N-terminal tail of the histone. Histone acetyl transferases (HATs) and histone deacetylases (HDAC) are the enzymes responsible of controlling the addition and removal of the acetyl group to histones, in an ATP-dependent manner (Verdone et al., 2005; Lakshmaiah et al., 2014). The addition of the acetyl group results in a charge change between histones and DNA. The acetyl group neutralizes lysine’s positive charge while unwinding the chromatin and hence reducing the affinity between histones and DNA. On the other hand, the removal of the acetyl group condenses the chromatin and promotes the binding of histones and DNA (Görisch et al., 2005). This usually occurs in histones H3 and H4 as they contain several lysine residues.

HDACs play a role in preparing the chromatin to promote the repair of DSBs via HR and NHEJ. One of the mechanisms in which this occurs is through the activation of potent poly (ADP-ribose) polymerse1 (PARP1), a protein abundantly present in the nucleus, that is responsible for post-translational changes by attaching a negatively charged polymer, poly (ADP-ribose) (PAR), to itself and multiple proteins. This activity is known as PARylation (Meter et al., 2016; Mao et al., 2011). PARP1 and the PAR chain signal for the recruitment of the nucleosome remodeling deacetylation (NuRD) complex, which consists of HDAC1, HDAC2, RBBP4, RBBP7, MTA1/2/3, MBD3/2 and CHD3/4, that are essential for DSB repair. HDAC1 and HDAC2 deacetylase target sites at histone H4, which stimulate the RNF8/RNF168-dependent ubiquitination at DSB, promoting repair through NHEJ (Verreault et al., 1998; Chou et al., 2010; Polo et al., 2010; Millard et al., 2016). It has also been reported that the acetylation/deacetylation of specific sites in both histones H4 and H2 can create a switch from NHEJ to HR through the regulation of 53BP1 binding at the DSB site (Tang et al., 2013; Chapman et al., 2013).

A recent player in the DSB repair pathway, COMMD4, has shown promise as a potential prognostic marker and therapeutic target in non-small cell lung cancer. The authors demonstrated that COMMD4 depletion resulted in the induction of mitotic catastrophe and apoptosis of non-small cell lung cancer cells (Suraweera et al., 2020). COMMD4 has additionally been shown to regulate chromatin remodeling at sites of DSBs (Suraweera et al., 2021). COMMD4 is initially recruited to sites of DSBs by hSSB1 and here COMMD4 functions to protect H2B from ubiquitination by the RNF20/40 E3 ligase complex. In undamaged cells, COMMD4 remains bound to H2B. However, upon the induction of DNA damage and subsequent phosphorylation, followed by disruption of the H2A-H2B dimer, COMMD4 preferentially binds to H2A. This switching of COMMD4 from H2B to H2A, enables RNF20/40 access to H2B and proceed with chromatin remodeling for DSB repair. Thus, highlighting the interplay between epigenetic regulatory mechanisms and DSB repair.

In addition to histone modifications, DNA itself can be modified by methylation. Methyl groups are added to the DNA molecule at specific sites known as CpG islands (Figure 3B). Methylation has the ability of changing the activity of a DNA segment without altering its sequence and is suggested to be the most stable of all epigenetic markers, contributing to more sustainable genetic changes. This epigenetic mechanism involves three players: the DNA, the enzyme (DNMTs) and cofactors and the S-adenosyl-l-methionine (SAM) of the cytosines at protected CpG (cystosine-phosphate-guanine sites, 5′-3′) sites of the genome (Lande-Diner and Cedar, 2005). DNA methylation occurs in approximately 60–90 CpG islands located at the promoter regions of the many genes. DNMTs are responsible for DNA methylation in early development. DNMTs obtain the methyl group from an activated S-adenosylmethionine (SAM) which leads to the release of S-adenosylhomocysteine (SAH) as a bi-product (Finkelstein, 1990; Mato et al., 1997). This allows for a cytosine structural change to 5-methylcytosine. Demethylation, comprises the involvement of human ten-eleven translocation (TET) enzymes. These enzymes are responsible of adding a hydroxyl group to the 5-methylcytosine, which leads to the formation of 5-hydroxymethil cytosine that is later transformed back into cytosine with the intervention of other TET enzymes during different pathways (Pastor et al., 2013; Cimmino et al., 2017). Hypomethylation and hypermethylation contribute to genomic instability and it is a characteristic present in cancer tumors. DNA methylation affects gene expression through a “writer,” “reader” and “eraser” system. The writer and eraser proteins are the ones in charge of creating or deleting genomic modifications, meanwhile, readers oversee the recognizing of such changes (Kass et al., 1997). DNA methylation allows for the permanent silencing of a gene allowing for the transcriptional machinery to focus on the essential genes needed for the expression and continuity of a differentiated phenotype. It has been shown that DNA methylation plays an important role in early somatic cell differentiation and may also play a role in DNA damage repair (Khavari et al., 2010). Studies have indicated that DSBs can induce hypermethylation and therefore downregulate gene expression. Similarly, DNA damage and repair can lead to an accumulation of aberrant DNA methylation (O’Haganet al., 2008). Additional literature suggests that a balanced intake of nutrients contributes to the maintenance of an effective DNA repair machinery through DNA methylation. For example, dietary folate deficiency is linked with an increased risk of cancer development through DNA damage, hypomethylation and through the inhibition of DNA methyltransferases (Steevens et al., 2011; Kadayifci et al., 2018; Ferrari et al., 2019). Similarly, it has been observed that cancer patients with a low vitamin C diet can lead to an acceleration in cancer progression (Cimmino et al., 2017; Sant et al., 2018; Gillberg et al., 2019). This is because vitamin C can enhance the activity of DNMTs. In terms of its influence in chromatin structure, high levels of methyl-CpG have been associated with transcriptional inactivity and nuclear resistance in endogenous chromosomes (Antequera et al., 1999).

#### Mechanisms of Histone Deacetylases and Their Inhibitors

HDACs are not redundant in function and have been classified into four groups, based on their homology to yeast. Class I includes HDAC 1, 2, 3 and 8 (yeast RPD3 deacetylase related) which are highly homologous in their catalytic sites and are often ubiquitously expressed in the nucleus. Class II includes HDAC 4, −5, −6, −7, −9 and −10 (yeast Hda1 related), they are usually found in the cytoplasm, but they can also be found in the nucleus. They share homology in the C-terminal catalytic domain and the N-terminal regulatory domain. Class III HDACs are also known as “sirtuins”, which enzymatic activity is NAD + dependent (Vaquero et al., 2007). Class IV HDACs (yeast Hda1 related) include HDAC11 and share conserved residues in the catalytic region with class I and II HDACs (Voelter-Mahlknecht et al., 2005).

**FIGURE 4:**
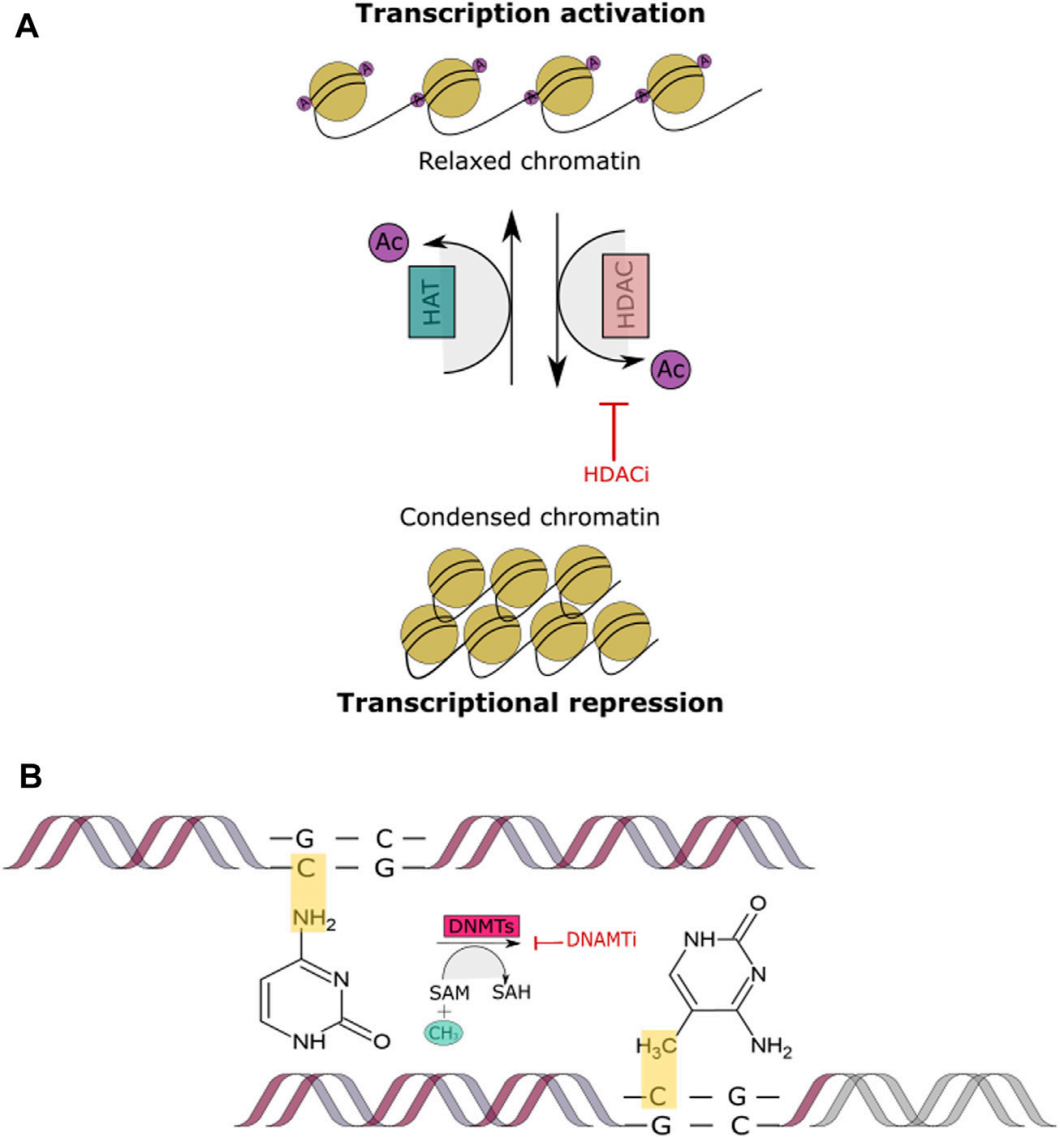
Histone Acetylation and DNA methylation. **(A)** This figure shows the acetylation mechanism of adding an acetyl coenzyme A (acetyl CoA) to the N-terminal tail of a histone through the HAT enzyme, leading to a relaxed chromatin. Conversely, histone deacetylation removes the acetyl CoA through the HDAC enzyme, leading to a condensed chromatin and transcriptional repression. When a HDACi is added the acetyl CoA group cannot be removed and therefore, the chromatin remains relaxed and transcription remains active. **(B)** This figure depicts DNA methylation process being blocked by a DNMTI. The inhibitor prevents the addition of the methyl group to the CpG island site in DNA, inhibiting transcriptional repression.

Due to the different roles in which HDACs are involved; histone deacetylase inhibitors (HDACi) are currently playing an important part in cancer therapy. As the name describes, their function is to inhibit HDAC activity. This occurs by promoting chromatin relaxation through acetylation and therefore, endorsing transcriptional activation (Figure 4A). HDACi have been classified into groups which include hydroxamates, cyclic peptides, aliphatic acids, benzamides and electrophilic ketones (Voelter-Mahlknecht et al., 2005). For example, Class I and II HDACs are often inhibited by trichostatin A (TSA), suberoylanilide hydroxamic (SAHA) and related compounds (Ruijter et al., 2003). HDACi have been reported to induce cancer cell cycle arrest, differentiation and cell death, reduce angiogenesis and modulate immune response (Eckschlager et al., 2017). In the context of DSB repair, one of the observed outcomes indicates that HDAC inhibition or knockdown leads to the downregulation of RAD51 or Mre11 of the HR pathway. Similarly, it has been demonstrated that inhibition of HDAC1/2/3 leads to high levels of acetylated Ku 70/80, decreasing its bonding affinity to the DSB ends and therefore decreasing DSB repair via NHEJ. It has additionally been shown that the use of HDACi can increase sensitivity to DSB inducing chemotherapeutics (Koprinarova et al., 2011; Zhao et al., 2017), which occurs through their ability to alter the expression of the most critical proteins involved in the DNA DSB repair pathways.

HDAC1, 2 and 3 are involved in the direct regulation of non-histone proteins that play a critical role in DSB repair pathways. This occurs via acetylation/deacetylation of proteins, such as Ku70. Studies have reported a histone acetylation-independent mechanism by which the HDAC inhibitors; trichostatin A, suberoylanilide hydroxamic acid, MS-275, and OSU-HDAC42, are able to sensitize prostate cancer cells to DNA damaging agents through the regulation of Ku70 acetylation (Chen et al., 2007). Similarly, it has been shown that during HR, ATM is required for DSB-induced RAD52 acetylation through HATs (p300/CBP) (Yasuda et al., 2018). Rad52 acetylation is important for RAD51 colocalization at the DSB site, therefore, it plays an intrinsic role in the HR repair pathway. It has also been found that human SIRT6-dependent CtIP deacetylation promotes DNA resection, a crucial step in DNA DSB repair by HR (Kaidi et al., 2010). These approaches by which HATs/HDACs lead to mechanisms such as cell sensitization and or the regulation of RAD52 acetylation have been recognized as promising targets for cancer therapy. The use of epigenetic agents can be quite complex. A study showed that the inhibition of HDAC1 and HDAC2 was consistent with a decreased survival of cells upon induction of DSB, suggesting that these lysine deacetylases could potentially promote DSB repair by removing histone marks at the DNA damaged site (Miller et al., 2010). Further studies have revealed the existence of a DNA DSB-induced monoubiquitination-to-acetylation switch on histone H2B, regulated through the SAGA complex, as well as higher-ordering signaling at HR repaired DSBs whereby histone H1 is evicted, while ubiquitin and 53BP1 accumulate over γH2AX domains (Clouaire et al., 2018).

#### Mechanisms of DNA Methyltransferases and Their Inhibitors

DNMTs are enzymes that interact directly with the chromatin through chromatin-associated proteins, which bind to the histone tails at specific unmethylated sites, e.g., ADD, PWWP domains, H3K4 (Zhang et al., 2010). They are part of a family consisting of a conserved set of DNA-modifying enzymes. DNMT1, DNMT2, DNMT3A, DNMT3B and DNMT3L are the five encoded human DNMTs from which only DNMT1, DNM3A and DNMT3B are canonical cytosine-5 that catalyze the addition of methyl groups to the DNA (Figure 4B). Whenever there is a dysregulation in the expression of genes that encode for DNA methylation there are also implications in the regulation of DNMT activity. These regulations can be affected by molecular interactions, post-translational modifications, alternative splicing and through gene loss and duplication (Li and Zhang, 2014; Robertson et al., 1999; Jeltsch and Jurkowska 2016; Aapola et al., 2000). These alterations often lead to the hypermethylation of tumors, however, the explanation behind such events still needs to be explored. In contrast to methylation of the CpG islands which leads to gene silencing, demethylation promotes gene activation. Studies have shown that DNMTi are able to reactivate tumor suppressor genes. In order to inhibit methylation, any of the three parts that comprise the catalytic pocket can be targeted, which is a promising approach for cancer treatment (Figure 4B) (Gros et al., 2015; Kim et al., 2012; Mair et al., 2014; Daskalakis et al., 2002).

### Histone Deactylase Inhibitors and DNA Methyltransferase Inhibitors as Epigenetic Drugs Used in the Clinic

Studies have shown that modulation of HAT and HDAC are promising approaches to treat malignant gliomas, T-cell lymphoma, multiple myeloma, breast cancer and other malignancies (Werner et al., 2017; Eyüpoglu and Savaskan, 2016). Understanding how these modulations work has helped improve cancer classification schemes, identify markers for early cancer detection and/or monitoring metastatic disease, improve therapy response and dictate prognosis.

HDACi and DNMTi are the most predominantly approved epigenetic drugs (epi-drugs) by the FDA (Tables 1,2). Preclinical studies have recently started testing DNMTi and HDACi in combination with immunotherapies and have shown promising clinical responses in cancers such as lung adenocarcinoma, myeloid-derived carcinomas, melanoma and lymphomas (Mazzone et al., 2017) (Tables 1,2).

**TABLE 1:** Most common clinically used histone deacetylase inhibitors that have been approved by the FDA or are currently undergoing clinical trials for the treatment of cancer.

HDAC inhibitor	HDAC class	Maximum phase of therapy	Cancer type	Status	FDA approval	DNA damage impact: Proteins regulated or involved/pathway impact/cellular response
Romidepsin	Cyclic tetrapeptide	Phase III	Peripheral T cell lymphoma	Active, not recruiting	No	DNA damage and apoptotic cell death through caspase activation; accumulation of DNA-RNA hybrids (R-loops); radiosensitiser; activation of ATM pathway, increased production of reactive oxygen species (ROS), decreased mitochondrial membrane potential Valdez et al., (2018); Miles et al., (2019); Paillas et al., (2020); Rossetti et al., (2021); Safari et al., (2021)
		Phase II	Cutaneous T-cell lymphoma; peripheral T-cell lymphoma; T-cell non-Hodgkin lymphoma	Completed	Yes	
		Phase I/II	Relapsed/refractory T-cell lymphoma; peripheral T-cell lymphoma; relapsed/refractory lymphoid malignancies; multiple myeloma, non- Hodgkin’s lymphoma; recurrent or metastatic triple negative breast cancer	Active, not recruiting	No	
Panobinostat	Hydroxamates	Phase III	Multiple myeloma	Completed	Yes	Pleiotropic antitumour effects and autophagy; induces clastogenicity, aneugenicity, oxidative damages and hypomethylation; increased G2/M arrest and production of ROS, enhanced proton-induced DNA damage A. Wilson et al., (2020); Choi et al., (2021); Al-Hamamah et al., (2019); Medon et al., (2017)
		Phase III	Acute myeloid leukemia; myelodysplastic syndromes	Completed	No	
		Phase II	Multiple myeloma; recurrent plasma cell myeloma; refractory/relapsed multiple myeloma; relapsed/refractory non-Hodgkin lymphoma; diffuse intrinsic pontine glioma	Active, not recruiting	No	
Mocetinostat	Benzamide	Phase II	Non-small cell lung carcinoma	Active, not recruiting	No	Potentially regulates RAD51 through HDAC2 in some cancers; maintains chromatin state; chemosensitizer; tumor suppression; increases tumor antigen presentation; cell cycle progression; suppresses cell proliferation; induces apoptosis through the upregulation of miR-31 (pro-apoptotic microRNA) (Briere et al., (2018); Q. Zhang et al., (2016b; Mondal et al., (2020); Headley et al., (2019); Shan et al., (2017); Yan and Efferth (2020)
		Phase I/II	Hodgkin lymphoma; lymphoma; relapsed/refractory hodgkin lymphoma; relapsed and refractory diffuse large B-cell lymphoma and follicular lymphoma	Active, not recruiting	No	
MS-275	Miscellaneous	Phase III	Advanced/metastatic breast cancer	Active, not recruiting	No	Inhibits RAD51/FANCD2 mediated HR; increases radiosensitization by prolongation of γH2AX Yao et al., (2018); Christmann and Kaina (2019); Kaina and Christmann (2019)
		Phase II	Renal cell carcinoma; male breast carcinoma, recurrent breast carcinoma; endometrial endometrioid adenocarcinoma; cholangiocarcinoma and pancreatic cancer; metastatic pancreatic cancer; metastatic uveal melanoma; bladder cancer; advanced or recurrent breast cancer	Active, not recruiting	No	
		Phase I/II	Epithelial ovarian cancer; peritoneal cancer; fallopian tube cancer; CNS tumor; solid tumor; non-small cell lung cancer; melanoma; mismatch repair-proficient colorectal cancer; clear renal cell carcinoma; metastatic kidney carcinoma; stage III, IV renal cell cancer; breast neoplasm	Active, not recruiting	No	
Abexinostat	Hydroxamates	Phase III	Renal cell carcinoma	Active, not recruiting	No	Regulates RAD51 (Kashyap et al., (2020)
		Phase II	Relapsed/refractory follicular lymphoma	Active, not recruiting	No	
Belinostat	Hydroxamates	Phase II	Peripheral T-cell lymphoma	Completed	Yes	Upregulates the expression of several genes in DNA damage pathway (PARP1, Gadd45a, Mpg); downregulates the expression of several genes involved in DNA damage pathway (Cdc25c, RAD 18, 51, 9, 1, TRP53, XRCC1); radiosensitizing through the induction of oxidative stress and DNA damage; interferes with mitotic spindle assembly; promotes stem cell differentiation and inhibits MYC pathways (García-Giménez et al.,; To et al., (2017); F. Chi et al., (2021; Marijon et al., (2018); Attia et al., (2018)
		Phase II	Unresectable/metastatic conventional chondrosarcoma; glioblastoma multiform of brain; T-cell leukemia-lymphoma	Active, not recruiting	No	
Valproic acid	Short-chain fatty acid	Phase IV	Seizure treatment in glioma	Completed	Yes	Upregulates gadd45a; radiosensitizer via increase of γH2AX phosphorylation; alters cell proliferation, cell survival, cell migration and hormone receptor expression; increases cell cycle arrest by increasing the expression of cyclin dependent kinase inhibitor (CDKN1A) Jang et al. (2020); Gao et al., (2020); Yan et al., (2021); Bhatti et al., (2021); Ding et al., (2020)
		Phase II	High-grade glioma; myelodysplastic syndromes	Active, not recruiting	No	
		Phase I/II	Solid tumors; acute myeloid leukemia	Active, not recruiting	No	
Vorinostat	Hydroxamates	Phase III	Multiple myeloma; relapsed/refractory cutaneous T-cell lymphoma	Active, not recruiting	No	Downregulates the expression of genes involved in DNA repair pathway (BIRP1, CDC25C, RAD proteins, TRP53, XRCC1); upregulates mRNA transcripts of repair genes implicated in DNA damage (Gadd45a, PARP1, BAX); induces chromosomal aberrations, oxidative damages, apoptosis and hypomethylation; decreases cellular viability and ROS (Singh et al., (2021); Sher et al., (2020); Zhang et al., (2020); Attia et al., (2020)
		Phase II	Cutaneous T-cell lymphoma	Completed	Yes	
			Breast cancer; neuroblastoma; adenomas in Cushing’s disease; cutaneous T-cell lymphoma/mycosis fungoides; myelodysplastic syndromes or chronic myelomonocytic leukemia	Active, not recruiting	No	
		Phase II/III	High grade glioma	Active, not recruiting	No	
		Phase I/II	Recurrent squamous cell head and neck cancer or salivary gland cancer; melanoma, skin neoplasms; multiple myeloma; advanced sarcoma; diffuse large B-cell lymphoma (stage II, III or IV); glioblastoma; glioblastoma multiforme; HIV-related diffuse large B-cell non-hodgkin lymphoma; acute myeloid leukemia in remission; myelodysplastic syndromes or acute myeloid leukemia	Active, not recruiting	No	
Nicotinamide	Sirtuins inhibitors	Phase III	Head and neck cancer; skin cancer	Completed	Yes	Represses genes involved in DNA damage and repair (FANCD2, BRCA1, RAD51; increases levels of phosphorylated DDR markers (γH2AX, pChk1 and p53) leading to cellular sensitivity (Pillay et al., (2021); Ogino et al., (2019); Magalhaes et al., (2021); Singh et al., (2021)
		Phase II	Non-melanoma skin cancer, squamous cell carcinoma, basal cell carcinoma; breast cancer metastatic, platinum resistant recurrent ovarian cancer; metastatic lung carcinoma; chronic myeloid leukemia	Active, not recruiting	No	
		Phase II/III	Non-small cell lung carcinoma	Active, not recruiting	No	

Source: U.S. National Library of Medicine, U.S. Food and Drug Administration, NIH Clinical Trial database: www.clinicaltrials.gov

**TABLE 2:** Most common DNA methyltransferase inhibitors that have been approved by the FDA or are currently undergoing clinical trials for the treatment of cancer.

DNMT inhibitor	DNMT class	Maximum phase of therapy	Cancer type	Status	FDA approval	DNA damage impact: Proteins regulated or involved/pathway impact/cellular response
5-Azacitidine	Nucleoside	Phase III	Continued treatment of acute myeloid leukemia and treatment of all subtypes of myelodysplastic syndrome	Completed	Yes	Cytotoxicity caused by genomic instability and DNA damage as a result of hypomethylation; reactivation of tumor suppressor genes (TSG); apoptosis through the reduction of MCL-1 expression levels (Guo et al., 2021; Guirguis, Liddicoat, and Dawson 2020; Goel et al., 2021; Zhou, Li, and Liu 2018)
			Acute myeloid leukemia; myelodysplastic syndromes	Active, not recruiting	No	
		Phase II/III	Acute myeloid leukemia or high-risk myelodysplastic syndrome	Active, not recruiting	No	
		Phase II	Advanced solid tumors; male breast carcinoma; recurrent breast cancer, stage IIIC breast cancer; stage IV breast cancer, triple negative breast carcinoma; neoplasms; pancreatic cancer; epithelial ovarian cancer; advanced/metastatic non-small cell lung cancer; prostate cancer; ovarian, primary peritoneal, or fallopian tube cancer; peripheral T-cell lymphoma; Chronic myeloid leukemia; relapsed/refractory acute myeloid leukemia or relapsed/high-risk myelodysplastic syndrome	Active, not recruiting	No	
		Phase I/II	Mutant myeloid neoplasm; solid tumors, gliomas; acute myeloid leukemia; myelodysplastic syndrome; non-Hodgkin lymphoma, multiple myeloma, lymphocytic leukemia; recurrent ovarian, fallopian tube or primary peritoneal cancer	Active, not recruiting	No	
Decitabine (analogues: 5-Aza-fluoro-2-deoxycytidine; zebularine)	Nucleoside	Phase IV	Acute myeloid leukemia	Active, not recruiting	No	Increases DSB frequency; reduces proliferation through PARP binding; invasion and adhesion; activation of tumor suppressor genes (VHL, CDKN2A, GATA4, MLH1) Sato, et al. (2017); Dellomo et al. (2019); Kashyap et al. (2020); Nigris et al. (2021)
		Phase III	Myelodysplastic syndromes (MDS) including myelomonocytic leukemia	Completed	Yes	
		Phase III	Acute myeloid leukemia; myelodysplastic syndromes	Active, not recruiting	No	
		Phase II/III	Acute myeloid leukemia or high-risk myelodysplastic syndrome	Active, not recruiting	No	
		Phase II	Non-small cell lung cancer; acute myeloid leukemia; leukemia; myelodysplastic syndromes	Active, not recruiting	No	
		Phase I/II	Advanced solid tumors; acute myeloid leukemia; acute myelogenous leukemia; diffuse large B cell lymphoma	Active, not recruiting	No	
MG98	Oligonucleotide	Phase I	Solid tumors	Completed	No	Cellular sensitization, growth inhibition concomitant with re-expression of TSGs P16ink4a and RUNX3 Beaulieu et al. (2004); Reu et al. (2004); Ramezankhani et al. (2021)
S110	Miscellaneous	Phase III	Acute myeloid leukemia; myelodysplastic syndromes, chronic myelomonocytic leukemia	Completed	No	Suggested to be a damaging variant of the NHEJ pathway through XRCC4; retards tumor growth Voorde et al. (2012); Singh et al. (2018)
		Phase II	Small cell lung cancer; myeloproliferative neoplasms; recurrent ovarian carcinoma, primary peritoneal or fallopian tube cancer; urothelial cancer; high-risk myelodysplastic syndrome	Active, not recruiting	No	
		Phase I/II	Advanced kidney cancer; recurrent ovarian, fallopian tube or primary peritoneal cancer	Active, not recruiting	No	

Source: U.S. National Library of Medicine, U.S. Food and Drug Administration, NIH Clinical Trial database: www.clinicaltrials.gov

Vorinostat was the first HDACi approved by the FDA in 2006 for the treatment of T-cell lymphoma. Seventy-four patients were part of the clinical trial from which 61 had at least stage IIB disease. The overall response rate (ORR) was 29.7% overall; 29.5% in stage IIB or higher patients. Median time to response in stage IIB or higher patients was 56 days. Median duration of response (DoR) was estimated to be ≥ 185 days. Median time to progression was 4.9 months overall and ≥9.8 months for stage IIB or higher responders. Overall, 32% of patients had pruritus relief. Adverse effects included diarrhea (49%), fatigue (46%), nausea (43%), and anorexia (26%); most were grade ≤2. Those grade ≥3 included fatigue (5%), pulmonary embolism (5%), thrombocytopenia (5%), and nausea (4%) (Olsen et al., 2007). Vorinostat clinical trials are ongoing for the treatment of other cancers such as breast cancer, high grade glioma and acute lymphoblastic leukemia (Table 1). This drug can be used by itself or in combination with other therapies such as narrowband UVB. This approach has been successful for the treatment of different types of cutaneous T-cell lymphoma (CTCL) (Geskin, 2007; Mann et al., 2007; Ragheb et al., 2017).

Vorinostat in combination with the chemotherapy drug, etoposide, is currently undergoing phase I/II clinical trials for the treatment of solid tumors and relapsed refractory sarcomas in pediatric patients (ClinicalTrials.gov Identifier: NCT01294670). It is also being tested in combination with pembrolizumab to treat patients with advanced lung cancer (ClinicalTrials.gov Identifier: NCT02638090). Valproate (valproic acid) was approved by the FDA in 2008 for seizure treatment in gliomas. It is currently undergoing clinical trials (phase I/II) in combination with neratinib (tyrosine kinase inhibitor) to treat patients with advanced solid tumors (ClinicalTrials.gov Identifier: NCT03919292). Romidepsin was approved in 2009 for the treatment of CTCL and in 2011 for the treatment of peripheral T-cell lymphoma (PTCL). Romidepsin is currently undergoing clinical trials for the treatment of cancers such as relapsed/refractory T-cell lymphoma and peripheral T-cell lymphoma (Table 1). Ongoing studies involving romidepsin in combination with tenalisib (PI3K inhibitor) are currently on phase I/II for the treatment of patients with relapsed/refractory T-cell lymphoma (ClinicalTrials.gov Identifier: NCT03770000).

Belinostat was approved by the FDA in 2014 for the treatment of peripheral T-cell lymphoma. The clinical trial was a single-arm, open-label, multicentre trial in relapsed or refractory peripheral T-cell lymphoma (PTCL) patients. One hundred and twenty-nine patients were involved in the trial (range, 29–81 years old) from which the majority of patients had stage III or stage IV disease. The overall response rate (ORR) was 25.8% with a complete response (CR) of rate of 13% and partial response (PR) rate of 18%. Among responding patients treated with belinostat, probability of maintaining response was 57.7% at 6 months, 48.8% at 1 year and 32.6% at 2 years. The probability of surviving and being progression free at 1 year was 19.3%. One hundred and thirteen patients out of 129 tolerated belinostat well, median treatment duration was 7 weeks. The adverse events occurred in 96.9% of patients being generally mild to moderate in severity. These included nausea (41.9%), fatigue (37.2%), and pyrexia (34.9%). Grade 3–4 thrombocytopenia occurred in only 7.0% (O’Connor et al., 2015). Belinostat is currently undergoing studies to be used in the clinic for unresectable/metastatic conventional chondrosarcoma; glioblastoma multiforme and T-cell leukemia-lymphoma (Table 1). Clinical studies on belinostat in combination with SGI-110 (guadecitabine/hypomethylating agent) are currently in phase II trials for the treatment of unresectable and metastatic conventional chondrosarcoma (ClinicalTrials.gov Identifier: NCT04340843).

Panobinostat, was approved by the FDA in 2015 and has shown to be effective against Multiple Myeloma. The clinical trial consisted of combining panobinostat, bortezomib and dexamethasone with placebo, bortzomib and dexamethasone. This was a multicentre, randomized, placebo-controlled, double-blind phase III trial of relapsed or relapsed and refractory multiple myeloma who were randomly assigned 1:1. Seven hundred and eighty-six patients participated in the study. The median follow-up was 6.47 months in the panobinostat group and 5.59 months in the placebo group. The median progression-free survival was significantly longer in the panobinostat group than in the placebo group (11.99 vs 8.08 months, *p* < 0.0001). At the time of the study the overall survival was not yet mature. Serious adverse responses were reported in 60% of the 381 patients in the panobinostat group and 42% of 377 patients in the placebo group. Common grade 3–4 laboratory abnormalities and adverse events included thrombocytopenia (67% panobinostat group vs 31% placebo group), lymphopenia (53 vs 40%), diarrhea (26 vs 8%), asthenia or fatigue (24 vs 12%) and peripheral neuropathy (18 vs 15%) (San-Miguel et al., 2014). Other studies suggest that panobinostat may also be effective against triple negative breast cancer, non-small cell lung cancer and head and neck squamous cell carcinoma (HNSCC) (Raedler, 2016; Suraweera et al., 2018) (Table1). Additionally, panobinostat in combination with carfilzomib (proteasome inhibitor) is currently in phase I/II clinical trials for the treatment of patients with relapsed/refractory MM (ClinicalTrials.gov Identifier: NCT01496118).

DNMTis can be nucleoside, non-nucleoside or oligonucleotide. Nucleoside DNMTis are integrated into the DNA and are prone to toxicity (e.g. 5-azacytidine, azacytosine and zebularine) (Table 2) (Zhou et al., 2002; Stresemann and Frank 2008; Gnyszka et al., 2013). On the other hand, non-nucleoside DNMTis are less toxic and usually more effective because they are not integrated into DNA (e.g., epigallocatechin-3-gallate EGCG, RG108 and procaine) (Y. C. Li et al., 2018; Rondelet et al., 2017; Zhang et al., 2016a). Oligonucleotides comprise antisense molecules such as MG98 (Davis et al., 2003) (Table 2). 5-Azacytidine (Vidaza) was the first DNMTi approved by the FDA in 2008 to be used in the clinic for the treatment of patients with myelodysplastic syndromes (MDS) (Table 2). In a phase III, international, multicentre, controlled, parallel-group, open-label trial, 358 patients with higher-risk myelodysplastic syndromes were randomly assigned 1:1 to receive azacytidine (*n* = 179) or conventional care (*n* = 179). With a median follow-up of 21.1 months the median overall survival was 24.5 months for the azacitadine group vs 15.0 months for the conventional care group. At 2 years the estimated overall survival was 50.8% for patients in the azacitadine group and 26.2% in the conventional care group (*p* < 0.0001). Peripheral cytopenias were the most common grade 3–4 adverse events for all treatments (Fenaux et al., 2009). Azacitidine is currently undergoing phase IV clinical trials in combination with HAG (Homoharringtonine, Cytarabine, G-CSF) regimen for the treatment of elderly patients with newly diagnosed myeloid malignancy (ClinicalTrials.gov Identifier: NCT03873311). It is also being studied in combination with the mutant p53 reactivating compound APR-246 (phase I/II) for the treatment of MDS and acute myeloid leukemia (ClinicalTrials.gov Identifier: NCT03588078). Another DNMTi known as decitabine (DACOGEN) has recently been approved by the FDA in combination with cedazuridine for the treatment of previously treated/untreated, *de novo* and secondary MDS as well as intermediate 1, 2 and high-risk International Prognostic Scoring System groups (FDA n. d.). Decitabine, alone, was initially approved in 2006 for the treatment of MDS. A total of 170 patients with MDS were randomized to receive either decitabine or best supportive care. Patients treated with decitabine achieved a significantly higher ORR (17%), including 9% CR, compared with supportive care (0%) (*p* < 0.001). Responses were durable (median, 10.3 months) and a trend toward a longer median time to acute myelogenous leukemia progression or death compared with patients who received supportive care alone was observed (Kantarjlan et al., 2006). Decitabine’s efficacy has led to continuous studies for the treatment of different cancers such as primary malignant neoplasm of ovary, metastatic renal cell carcinoma and non-small cell lung cancer (Table 2). Hydralazine is a vasodilator initially approved by the FDA in 1997 for the treatment of high blood pressure and heart failure. However, recent studies have shown that it also acts as a DNMTi by inducing caspase-dependent apoptotic cell death in p53-mutant leukemic T lymphocytes (Ruiz-Magaña et al., 2016).

Despite, the promising outcomes of these epigenetic mechanisms in cancer patients, the anti-tumour activity achieved by HDACi and DNMTi are still limited. For instance, an alternative approach has been the use of combination therapy. Two or more therapeutic agents that individually produce similar or additive effects will often display enhanced efficacy, referred to as synergy, when given in combination (e.g., drug 1 + drug 2 = synergy). In this review we will mainly focus on the combination of HDACi and/or DNMTi together with DNA repair inhibitors and/or immune checkpoint inhibitors. The purpose behind this combination treatment approach is to target the blocking of several key pathways. Thus, to reshape the tumor microenvironment and potentially obtain a synergistic ani-tumour response that would be greater than that predicted by their individual potencies (Zeng et al., 2016; Villanueva et al., 2020; Zhou et al., 2018).

#### Histone Deactylase Inhibitors and DNA Methyltransferase Inhbitors in Combination With DNA Repair Inhibitors in the Clinic

The advent of PARP inhibitors has pinpointed DNA repair inhibitors as predominant targets for cancer therapy (Tangutoori et al., 2015). Olaparib (Lynparza), is a PARP inhibitor (PARPi) that targets the DNA damage response as a single agent for the treatment of breast and ovarian cancers in patients harboring BRCA1 or BRCA2 germline mutations (Kim et al., 2015). PARP anti-tumour activity is based on inducing defects in genes/pathways leading to genomic instability. PARPi induce apoptosis caused by the aggregation of DNA damage which favors the flow of T-cells into the tumor microenvironment, triggering the upregulation of PD-1/PD-L1 pathway. At present there are several clinical trials underway combining HDACi in combination with olaparib. A phase I clinical trial combining olaparib and vorinostat, busulfan, gemcitabine and melphalan with or without rituximab, has started for patients suffering from refractory lymphomas (ClinicalTrial.gov identifier: NCT03259503). There are additionally several clinical trials underway combining olaparib and entinostat for the treatment of ovarian carcinoma, peritoneal carcinoma fallopian tube carcinoma (ClinicalTrial.gov identifier: NCT03924245) and olaparib in combination with vorinostat for the treatment of relapsed, refractory and/or metastatic breast cancer (ClinicalTrial.gov identifier: NCT03742245).

Other approaches include a study conducted by (Kim et al., 2012), which suggests that DNMTi are able to induce radiosensitivity in a cell line model with A549 and U373MG cells together with an extended activity of γH2AX, which is believed to be achieved through DNA repair inhibition. However, more studies are needed to identify other additional mechanisms that can also be associated with radiosensitivity and to confirm the synergistic effects on radiosensitivity with other epigenetic drugs such as HDACi. It is expected that further investigation on this method will help determine whether the combination of DNMTi and radiation has potential as a future clinical approach for cancer treatment. Another approach involves using DNMTi in multiple myeloma cells through an ataxia telangiectasia and Rad3-related protein mediated manner that induces DNA DSBs, leading to apoptosis. (Kiziltepe et al., 2007). This study suggests significant relevance into pursuing more in-depth clinical trials involving 5-AzaC alone and in combination with other chemotherapy drugs for the treatment of multiple myelomas (Table 2). More recent examples of drug combinations are, the dual DNMTi and HDACi 208, which has shown to instigate antiproliferative activity against histiocytic lymphoma (U937) cells (Zhou et al., 2018). This occurs by inducing G1 cell cycle arrest and apoptosis through the upregulation of CDK inhibitor p16, combined with the downregulation of cyclin-dependent kinases and their activators. Proteome and bioinformatic analyses showed that 208 inhibitor combinations affected the expression of a series of proteins involved in DNA repair. Similarly, PARPi has been studied in combination with DNMTi (e.g. guadecitabine or 5-azacitidine) with the purpose of being able to resensitize tumors to primary therapies or reprogramming DNA damage repair responses in cancers such as breast, ovarian and non-small cell lung cancers (Abbotts et al., 2019; Zhou et al., 2018; Muvarak et al., 2017).

Previous literature also indicates that CRISPR/dCAS9 can induce histone acetylation/deacetylation and methylation by catalyzing direct covalent modifications or via the recruitment of complexes that mediate such mechanisms (Tang et al., 2019; Thakore et al., 2015; Hilton et al., 2015). Similarly, DNA methylation/demethylation mechanisms can be programmed for the methyl groups to be added or removed from specific CpG island sites using CRISPR/dCas9. This epigenetic editing approach has been under continuous investigation as it proves to be more effective than modifications previously attempted by ZINC finger nucleases and TALENs modifications (Zhou et al., 2018; Thakore et al., 2015; Hilton et al., 2015; Zhang et al., 2017; Chi et al., 2021b). The use of CRISPR/dCas9 is a powerful candidate to manipulate the expression of therapeutic target genes, via epigenetic mechanisms, in cancer cells. (Jiang et al., 2015; Momparler et al., 2017; Wang et al., 2018).

#### Histone Deactylase Inhibitors and DNA Methyltransferase Inhibitors in Combination With Immune Checkpoint Inhibitors

Immune checkpoint inhibitors (ICis) are one of the most recent effective methods at reactivating anti-tumour responses in immune-oncology. They fulfill the role of keeping effector T-cells active in order to fight tumor cells. The first checkpoint inhibitor to be approved by the FDA was ipilimumab (targeting T-lymphocyte antigen-4, CTLA-4) for the treatment of melanoma patients (Hodi et al., 2010; Robert et al., 2011). Other ICis that have already been approved to be used in treatment are pembrolizumab and nivolumab as well as, atezolizumab, durvalumab and avelumab, used for the treatment of different carcinomas including metastatic melanoma, non-small cell lung cancer, renal cell carcinoma and neck squamous carcinoma (Kim, 2017; Syed, 2017; Horn et al., 2018; Ferris et al., 2016; Reck et al., 2016; Khoja et al., 2015). The latest monoclonal antibody approved by the FDA is cemiplimab for the treatment of metastatic cutaneous squamous cell carcinoma (Markham and Duggan, 2018). There have been several studies of HDACi and DNMTi in combination with ICis as an innovative approach in immunotherapy. Studies have shown that bladder tumors carry upregulated levels of HDACs. Pre-clinical trials are currently ongoing for using the HDACi, romidepsin and SAHA, in combination with HR-DNA repair genes and PARPi for the treatment of bladder cancer (Criscuolo et al., 2019). Additionally, DNMTi 5-aza-2′-deoxycytidine is currently undergoing trials to be used together with CTLA-4 for the treatment of mammary carcinoma and mesothelioma (Covre et al., 2015). PD-1/PD-L1 ICis have also been commenced in combination with alterations of DDR genes to treat urothelial carcinoma. It is expected that further studies involving HDACi, DNMTi and ICis will reveal novel ways of targeting genes involved in DDR, that can potentially be used as personalized immunotherapies (Daver et al., 2019; Gray et al., 2019).

#### Histone Deactylase Inhibitors and DNA Methyltransferase Inhibitors: Challenges to Overcome

Often, a single approach, such as adjuvant chemotherapy, is not effective in every patient and therefore leads to disease recurrence (Mamounas et al., 2017). Combination therapy is an approach designed to reinvigorate a drug’s effect against a specific type of cancer, however, this path is also not always a safe bet. For example, the clinical use of atezolizumab in combination with paclitaxel protein-bound (abraxane) has been approved by the FDA for the treatment of metastatic triple negative breast cancer (mTNBC) in adult patients expressing PD-L1 (Narayan et al., 2020). The FDA has raised awareness about recent clinical studies showing that atezolizumab + paclitaxel combination has no effect in previously untreated inoperable locally advanced or mTNBC (FDA 2020b). It would therefore be recommended that abraxane should not be replaced with paclitaxel in clinical practice. Consequently, these results will require further testing and the potential update of current prescribing information. It is also well known that cancer cells develop drug resistance and therefore, cells can develop DNMTi and HDACi resistance (Maeda et al., 2018). This can lead to an increase or decrease in activity of important pathways such as HR and NHEJ. Some epi-drugs such as nucleoside DNMTi, are introduced into the DNA and have a toxic effect and future research should focus on finding epi-drugs that are more effective and less toxic. Similarly, it can be challenging to identify an epigenetic target that remains stable when tested *in vivo*. The emergence of nanotechnology in cancer therapy has shown to be an encouraging strategy to enhance the effectiveness of HDACi (Tangutoori et al., 2015).

CRISPR/dCAS9 is a novel promising approach to achieve programmable histone modifications and DNA methylation. However, this mechanism is still in its early stages and it requires further research before it can be used in the clinic as an epigenetic therapy. Currently, there are still risks of off-target effects, and potential secondary effects caused by unintended factors (Tang et al., 2019; Zhang et al., 2017; Thakore et al., 2015; Hilton et al., 2015). Additionally, when not used appropriately, CRISPR is prone to non-specific binding. Regardless of the mechanism, if successful, an epigenetic drug may be effective in one type of cancer but not in others. This means that it will require further clinical studies. It is also important to consider that the effect of an epigenetic change may vary in different phases of the trial.

## Conclusion

DSBs are the most cytotoxic type of DNA backbone damage. In response to this genetic lesion, cells have evolved to recognize the damage and signal for DNA DSB repair mechanisms. Failing to repair DNA via HR or NHEJ pathways can lead to cancer and/or tumorigenesis. Investigating how cancer works from an epigenetic perspective has helped improve cancer classification schemes, identify markers for early cancer detection and/or monitoring metastatic disease, improve therapy response, dictate prognosis as well as helping in identifying epigenetic patterns associated to a cell’s transcriptional activity. DNMTi and HDACi have been shown to have positive effects in cancer treatment, especially when combined with traditional therapies or other epigenetic drugs. However, epigenetic drugs are just at the beginning of their apogee and there are still many factors to consider. Attention must be focused in finding epi-drugs that are more effective and less toxic; it is challenging to identify epigenetic targets that remain stable when tested *in vivo*. The CRISPR/dCAS9 approach to program the addition/removal of methyl groups still needs to be fine-tuned in terms of specificity. There are challenges in identifying epigenetic targets that remain equally effective in a type of cancer across all clinical trial phases. Taken together, epigenetic treatments are promising independent, combination treatment and potential personalized treatments in cancer therapy.
